# The Antitoxin Treatment of Diphtheria

**Published:** 1901-04-20

**Authors:** J. Odery Symes

**Affiliations:** Assistant Physician to the Hospital


### Hospital Clinics and Medical Progress.

### THE ANTITOXIN TREATMENT OF DIPHTHERIA.

A Clinical Lecture delivered at the Bristol General Hospital by J. Odery Symes, M.D., Assistant Physician to the Hospital.

Before entering upon the subject of the serum treatment of diphtheria, it will be well to review in brief the principles of general serum therapeutics. Behring was one of the first to point out that by injecting into horses increasing small doses of diphtheria toxins the animals could be made insusceptible to large doses of the poison or of the bacteria.

The next discovery was that the blood of such insusceptible animals, if injected into other animals, had the power of also rendering them immune; and, finally, that the blood, if injected into animals already suffering from the specific (diphtherial) infection, had a curative action. Applying these principles to diphtheria in human beings it was claimed that the serum of a horse immunised against diphtheria toxin would have a curative action if injected into persons suffering from diphtheria. This action is not a simple chemical one. The toxin in the sufferer's blood does not unite with the antitoxin and form an inert compound body.

In the case of faucial diphtheria the toxin is elaborated by the bacilli in the membrane on the throat, and by means of the lymphatics and blood-vessels reaches the general circulation and is brought into contact with the tissue cells. The proteid molecule of these cells consists of a closely-welded nucleus to which are attached other complex radicles called "side-chains." The toxin unites with these side-chains, alters them, and so alters the original molecule. To replace these side-chains new ones are formed, these become free in the circulation and meet fresh toxin and unite with it. The side-chains are, in fact, antitoxin, and in an individual, immunity to a given disease means absence of side-chains; high susceptibility the possession of many suitable side-chains. A given toxin can only unite with side-chains of a given and corresponding structure. In administering antitoxin to a case of diphtheria, therefore, we are but pouring into the general circulation a supply of side-chains (obtained from the horse) in order to neutralise the diphtheria toxin which is present in the system. The patient too, is manufacturing antitoxin from his own cells, but our fear is lest the supply be insufficient, and the toxins getting the upper hand lead to a fatal issue. The antitoxin introduced does not kill the baccilli; indeed, the Klebs-Löffler bacillus will live in diphtheria antitoxin, but in some way or other the bacterial activity is checked, as is evidenced by the fact that the membrane does not spread, and the administration of antitoxin is not required beyond the first three days.

From the preceding statements two points are evident. 1. Antitoxin to be of service must be administered in large doses. 2. It must be given early, before the diphtheria toxin has had time to destroy the proteid molecule, and before the toxin is present in overwhelming quantity. With regard to the second point I may quote to you the figures of the Metropolitan Asylums Board Hospitals. In pre-antitoxin days, of cases treated on the first day of the disease 22·5 per cent. died; second day, 27 per cent.; third day, 29·4 per cent.; fourth day, 31 per cent. Since the use of antitoxin the figures have been—first day, 4·7 per cent.; second day, 12·8 per cent.; third day, 17·7 per cent.; fourth day, 22·5 per cent. Tonkin gives very similar results.

| | Per cent. |
|---|---|
| First day ... ... ... ... ... ... | 0 |
| Second day ... ... ... ... ... | 5 |
| Third day... ... ... ... ... ... | 11 |
| Fourth day ... ... ... ... ... | 24 |
| Fifth day... ... ... ... ... ... | 32 |

Our views as to the dosage of antitoxin have undergone considerable modification of recent years. Formerly an initial dose of 1,000 units was given followed in succeeding days by doses of 500 units. At the present time very much larger doses are administered. In a severe case, as judged by the extent of the membrane and the gravity of the constitutional disturbance, the dosage should be as follows:—An initial dose of 4,000 units repeated every six hours during the first day, and doses of 2,000 units on each succeeding day until the membrane has cleared. In mild cases the initial dose of 4,000 should be followed in twelve hours time by a second of 2,000 units, and on subsequent days, provided progress is satisfactory, doses of 1,000 units should be injected. With this drug, as with any other, the effect has to be watched and the dose graduated according to the effect produced. An objection to large doses of antitoxin was formerly that they entailed the injection of large amounts of fluid which caused considerable pain and discomfort. Such an objection should not hold good to-day. A good antitoxin should contain at least 2,000 units in 5 ccs of fluid and some are made of double the strength, so that in no case is it necessary to inject more than 20ccs. (about half an ounce) Manufacturers of antitoxin do not pay sufficient attention to this matter of concentration, and if we examine four of the antitoxins which are most used at the present day (A.B.C.D.), we find the following wide variations:—

A. 5ccs. contain 500 or 1,000 units,
B. 5ccs. „ 1,500 or 2,000 units,
C. 5ccs. „ 2,000 or 4,000 units,
D. 5ccs. „ 2,000 units.

Not less widely divergent are the prices quoted. For concentrated sera the prices quoted for the above are:—

A. 9s. per 2.000 units.
B. 3s. 2d. „ „
C. 2s. 6d. „ „
D. 5s. „ „

This matter is one of great importance, for diphtheria is most prevalent amongst the poor, and if we suppose as small a quantity as 8,000 units to be given, then with the most expensive of these sera the treatment will cost thirty-six shillings, and with the cheapest ten shillings.

It is difficult to understand such wide differences in price and concentration, for the value of the "unit" is apparently the same in all cases, namely, that amount of serum which will completely neutralise ten lethal doses of diphtheria toxin when injected into a 260 gramme guinea-pig.

It is eminently desirable that all antitoxins sold should be examined and certified by some disinterested central body, such as the Conjoint Colleges of the Physicians and Surgeons. In this way their efficacy would be guaranteed and the treatment put upon a sounder basis. At such a laboratory too the serum would be tested for accidental bacterial contamination"; a most necessary precaution in view of the disastrous consequences following the distribution in Italy of diphtheria antitoxin containing tetanus bacilli.

It is a matter of considerable difficulty to form a correct estimate of the value of the antitoxin treatment of diphtheria. One's own personal experience is too limited; cases differ from one another very widely, and the type of disease varies greatly from time to time. Most clinical observers however agree that under the administration of antitoxin the membrane separates more readily, tracheotomy is less seldom called for, and when performed is more successful.

Nor do the reports of a big institution such as our own, the Bristol General Hospital, afford much assistance in forming an opinion. In the nine years preceding the administration of antitoxin (1886-1894) there were admitted:—

16 cases of diphtheria with 6 deaths (37·5 per cent.).
77 cases of laryngitis with 11 deaths.
24 cases of croup with 3 deaths.
43 cases of tonsilitis with no death.

From 1895–1899 there were admitted:—

29 cases of diphtheria, with 9 deaths (31 per cent.).
29 cases of laryngitis, with 4 deaths.
32 cases of tonsillitis, with 1 death.

Such figures prove nothing at all except that our nomenclature of disease has undergone and is undergoing a marked change. Croup ceases to figure after 1891, and laryngitis and tonsillitis are dwindling each year.

The records of our large municipal fever hospitals are likely to give us more accurate information, for only cases certified as diphtheria are treated, and the practitioner's diagnosis is subject to supervision by the medical superintendent. Take the case of the Metropolitan Asylums Board. From 1888–1894, previous to the use of antitoxin, there were admitted 11,598 cases of diphtheria, and of these 3,516, 30·3 per cent., died. From 1894–1898, 20,382 cases were treated, and of these 3,747, 18·4 per cent., died. The reduction in the case mortality has thus been 12 per cent. since the introduction of the new treatment. These figures, however, cannot be accepted as a true statement of the case, for latterly mild cases, only detected by recent methods of bacteriological examination, have been admitted in increasing numbers, and these will considerably diminish the mortality rate.

Laryngeal cases and cases requiring tracheotomy are, however, probably as severe now as they were before the days of antitoxin, and statistics relating to these are little likely to be vitiated by bacteriological examination. These, then, should give us the most reliable information as to the value of the treatment, and it is interesting to note that the recovery rate in such cases is now double that of pre-antitoxin days.

If we examine the records of our own city of Bristol, we find that in the five years preceding the introduction of antitoxin, to every 100,000 inhabitants there were 44·8 notifications of diphtheria with a case mortality of 34·5 per cent. In the five years following the introduction of the antitoxin treatment the notification rate was 80·7 per 100,000, and the case mortality 17·4 per cent. There has then been in this city a reduction of nearly 50 per cent. since antitoxin came into general use. This reduction in the case mortality of diphtheria is common to other cities, such as Edinburgh (7 per cent.), Glasgow )7·9 per cent.), and London (8·8 per cent.). Too much reliance, however, must not be placed on these statistics, for they are open to objections that have been already mentioned, namely: altered type of disease, and inclusion of slight cases detectable only by bacteriological examination. If we attribute the fall in mortality in these cities to the antitoxin treatment, how shall we account for the fact that no such fall in case mortality has been noted in Birmingham, Liverpool, and Manchester? The success of the antitoxin treatment has been greater abroad than in this country, where the reduction in the death-rate may be estimated at about 44 per million. In Italy there has been a reduction of over 100 per cent., in Berlin 50 per cent., Paris 80 per cent., Chicago 28 per cent., New York 20 per cent.

We see then that the reduction in the case mortality of diphtheria since the introduction of this remedy has been general, and that it is not confined to any one particular type of the disease. It may roughly be estimated as a reduction of 20 per cent. To some extent this reduction may be accounted for by the fact that since the introduction of bacteriological methods of diagnosis, mild cases which formerly escaped detection now come under treatment, and generally diagnosis is made at an earlier period of the disease. Taking these facts into consideration, however, there would appear to be no doubt that in antitoxin we have a valuable therapeutic agent for the treatment of diphtheria, but in order to ascertain the exact value of the remedy we need to pay closer attention in the future to careful clinical observation, accurate dosage, and reliable statistics.

